# L-NAC and L-NAC methyl ester prevent and overcome physical dependence to fentanyl in male rats

**DOI:** 10.1038/s41598-024-59551-0

**Published:** 2024-04-20

**Authors:** James N. Bates, Santhosh M. Baby, Paulina M. Getsy, Gregory A. Coffee, Yee-Hsee Hsieh, Zackery T. Knauss, Albert Dahan, Jason A. Bubier, Peter M. MacFarlane, Devin Mueller, Stephen J. Lewis

**Affiliations:** 1 Department of Anesthesiology, University of Iowa Hospitals and Clinics, Iowa City, IA USA; 2 Section of Biology, Galleon Pharmaceuticals, Inc, Horsham, PA USA; 3 Department of Pediatrics, Division of Pulmonology, Allergy, and Immunology, Case Western Reserve University, Cleveland, OH USA; 4 Division of Pulmonary, Critical Care and Sleep Medicine, Case Western Reserve University, Cleveland, OH USA; 5 Department of Biological Sciences, Kent State University, Kent, OH USA; 6 Department of Anesthesiology, Leiden University Medical Center, Leiden, Netherlands; 7 Jackson Laboratories, Bar Harbor, ME USA; 8 Department of Pharmacology, Case Western Reserve University, Cleveland, OH USA; 9 Functional Electrical Stimulation Center, Case Western Reserve University, Cleveland, OH USA; 10 Department of Pediatrics, Division of Pulmonology, Allergy and Immunology, School of Medicine,, Case Western Reserve University, 10900 Euclid Avenue, Cleveland, OH USA; 11 Present Address: Atelerix Life Sciences Inc., 300 East Main Street, Suite 202, Charlottesville, VA USA; 12 Present Address: Translational Sciences Treatment Discovery, Galvani Bioelectronics, Inc, 1250 S Collegeville Rd, Collegeville, PA USA

**Keywords:** Fentanyl, Physical dependence, N-acetyl-L-cysteine, N-acetyl-L-cysteine ethyl ester, Naloxone, Withdrawal phenomena, Drug discovery, Systems biology, Medical research

## Abstract

N-acetyl-L-cysteine (L-NAC) is a proposed therapeutic for opioid use disorder. This study determined whether co-injections of L-NAC (500 μmol/kg, IV) or its highly cell-penetrant analogue, L-NAC methyl ester (L-NACme, 500 μmol/kg, IV), prevent acquisition of acute physical dependence induced by twice-daily injections of fentanyl (125 μg/kg, IV), and overcome acquired dependence to these injections in freely-moving male Sprague Dawley rats. The injection of the opioid receptor antagonist, naloxone HCl (NLX; 1.5 mg/kg, IV), elicited a series of withdrawal phenomena (i.e. behavioral and cardiorespiratory responses, hypothermia and body weight loss) in rats that received 5 or 10 injections of fentanyl and similar numbers of vehicle co-injections. With respect to the development of dependence, the NLX-precipitated withdrawal phenomena were reduced in rats that received had co-injections of L-NAC, and more greatly reduced in rats that received co-injections of L-NACme. In regard to overcoming established dependence, the NLX-precipitated withdrawal phenomena in rats that had received 10 injections of fentanyl (125 μg/kg, IV) were reduced in rats that had received co-injections of L-NAC, and more greatly reduced in rats that received co-injections of L-NACme beginning with injection 6 of fentanyl. This study provides compelling evidence that co-injections of L-NAC and L-NACme prevent the acquisition of physical dependence and overcome acquired dependence to fentanyl in male rats. The higher efficacy of L-NACme is likely due to its greater cell penetrability in brain regions mediating dependence to fentanyl and interaction with intracellular signaling cascades, including redox-dependent processes, responsible for the acquisition of physical dependence to fentanyl.

## Introduction

There are many pressing issues facing clinicians treating people with opioid use disorder (OUD) and substance use disorder (SUD)^1,2^. The key issue related to treating pain involves how to provide μ-opioid receptor (μ-OR) agonist-induced analgesia without eliciting euphoria, physical dependence, addiction and enhanced physical and psychological pain/distress states (i.e., hyperalgesia)^3^. With respect to treating moderate to severe OUD, important issues are (1) how to manage the often severe opioid withdrawal, since current to manage opioid withdrawal have major weaknesses; (2) how to manage patients having gone through withdrawal who need medication to block euphoria and/or physical dependence, since available medications elicit both strengths and weaknesses; and (3) how to avoid opioid euphoria and/or physical dependence in patients with moderate to severe OUD who are actively sober, but need opioid analgesia^1–3^. A fundamental need in treating addiction to opioids, psycho-stimulants, alcohol, cannabinoids or benzodiazepines is to provide therapeutics that block the dopamine surge-induced euphoria produced by these brain-reward drugs^4^. Moreover, drugs that effectively modulate the actions of opioids in order to improve their safety and analgesic profile are lacking. The therapeutics should (1) not interfere with or promote opioid analgesia, (2) prevent acquisition of physical dependence and psychological addiction to opioids, (3) block opioid-induced respiratory depression (OIRD), and (4) stop the development of hyperalgesia^5^. Taken together, it is clear that there is an urgent need to develop therapeutics that prevent or overcome addiction/dependence to opioids by mechanisms other than modulating opioid-receptor (ORs).

Trivedi et al. provided evidence that morphine induced dependence/addiction may be due to redox-based changes in global DNA methylation and retrotransposon transcription via the inhibition of excitatory amino acid transporter type 3 (EAA3)-mediated uptake of cysteine into brain neurons^6^. Possible mechanism(s) from relevant studies of Trivedi et al.^6^ involve (1) morphine attenuation of L-cysteine uptake into neurons by G-protein-dependent decreases in EAA3 function, (2) subsequent reductions in cell concentrations of L-cysteine, L-glutathione and methylation index (SAM/SAH ratio, S-adenosyl-methionine/S-adenosyl-homocysteine), (3) reduced methylation of global CpG (regions of DNA where a cytosine nucleotide is followed by a guanine nucleotide in linear base sequence along the 5′ → 3′ direction) and decreased CpG methylation of long interspersed nuclear element—1 (LINE-1) retrotransposon regulatory regions, and (4) activation of transcription of previously silenced LINE-1 gene (see Fig. 5 of Trivedi et al.^6^). Based on this current research, we propose that N-acetyl-L-cysteine (L-NAC) may be a compound of interest to modulate opioid dependence because L-NAC enters both central and peripheral cells upon systemic injection or oral ingestion to increase the intracellular concentrations of L-cysteine and L-glutathione, and to provide reducing equivalents that exert signaling actions^7–12^. Additionally, Trivedi and Deth^13^ suggested that redox-based epigenetic status in drug addiction contributes to gene priming, and provided rationale for metabolic intervention including administration of L-NAC. Moreover, further studies found that L-NAC (1) reduced extinction responding and induced enduring decreases in cue- and heroin-induced drug-seeking^14,15^, (2) improved oxidative-stress imbalance within the hippocampus of morphine-treated rats^16^, (3) ameliorated the morphine-induced oxidative stress-dependent macrophage apoptosis^17^, (4) decreased remifentanil-induced postoperative hyperalgesia by inhibiting matrix metalloproteinase-9 in dorsal root ganglia^18^, (5) modulated the ability of δ-OR signaling to generate reactive oxygen species mediating intermittent hypoxia-induced protection of canine myocardium^19^, (6) overcame the adverse effects of fentanyl infusion on ventilatory parameters, Alveolar-arterial gradient (i.e., the index of alveolar gas exchange) and arterial blood-gas chemistry^20^, (7) ameliorated mitochondrial dysfunction in ischemia/reperfusion injury^21^ and (8) protected the kidneys from extra-corporeal lithotripsy-induced injury^22^. Furthermore, in humans and human cells, L-NAC has shown efficacy in (1) treating methamphetamine dependence^23^ and craving in SUD (Supplemental Table 1)^24,25^, (2) modulating the cysteine redox proteome in neuro-degenerative diseases^26^ and overcoming the down-regulation of anti-oxidant genes^27^, (3) acting as a pre-emptive drug to ameliorate post-operative pain^28^, (4) providing a new prominent approach for treating psychiatric disorders^29^, and (5) preventing/ameliorating peripheral neuropathies^30^.

Unfortunately, the poor cell penetrability of L-NAC is a barrier to its potential as a clinical therapeutic^28–30^. Luckily, a variety of cell-permeable analogues of L-NAC, including L-NACme, have been used in many patient circumstances (Supplemental Fig. 1 shows the structure)^31^. The rapid conversion of L-NACme to L-cysteine and L-glutathione in cells^31^ would possibly overcome the loss of the L-thiols elicited by opioids and restore biochemical/redox signaling including the potential formation of S-nitrosylated analogues of these thiols, such as S-nitroso-L-NACme^32^ and S-nitroso-L-cysteine, which blocks the respiratory depression induced by fentanyl or morphine^33,34^. Whether L-NAC or L-NACme attenuate acquisition of addiction/dependence to fentanyl has not been determined. Numerous administration/dosage paradigms have been employed to develop addiction to fentanyl in male and female rats including multiple injection protocols^35^. In the present study, we determine (1) whether co-injections of L-NAC or L-NACme diminish the development of acute physical dependence elicited by injections of fentanyl in male Sprague Dawley rats, as assessed by the withdrawal responses elicited by injection of the μ-OR antagonist, naloxone HCl (NLX), and (2) whether co-injections of L-NAC or L-NACme to rats that are physically-dependent on fentanyl could overcome this dependence, again assessed by the expression of the NLX-precipitated withdrawal responses. The fentanyl dose-regime consisting of twice daily intravenous injections of 125 μmol/kg was based on daily doses used by others^35^. The behavioral, cardiorespiratory, thermoregulatory and body weight changes were chosen on the basis of previous studies determining the scope and types of withdrawal phenomena^36^.

## Materials and methods

### Permissions, rats, and surgical procedures

All studies were performed as per the NIH Guide for Care and Use of Laboratory Animals (NIH Publication No. 80-23) revised in 1996, and ARRIVE (*Animal Research: Reporting of *In Vivo* Experiments*) guidelines (http://www.nc3rs.org.uk/page.asp?id=1357). All protocols involving rats were approved by the Animal Care and Use Committees of the University of Virginia, Case Western Reserve University and *Galleon Pharmaceuticals*. Adult male Sprague Dawley rats (n = 486) were purchased from *Harlan Industries* (Madison, WI, USA) and were given five days to recover from transportation before being subjected to surgeries. Fentanyl citrate, L-NAC and L-NACme powders were obtained from *Sigma-Aldrich* (St. Louis, MO, USA) and divided into 100 mg amounts under N_2_ gas and stored at 4 °C. Solutions of L-NAC and L-NACme (dissolved in saline and brought to pH 7.2 with 0.1 M NaOH at room temperature) were prepared immediately before use. Naloxone HCl (*Sigma-Aldrich*, St. Louis, MO, USA) was dissolved in normal saline. All arterial and venous catheters were flushed with 0.3 ml of phosphate-buffered saline (0.1 M, pH 7.4) 3–4 h before commencement of the study. All studies were done in a room with relative humidity of 49 ± 2% and temperature of 21.4 ± 0.2 °C. The numbers of rats and body weights for each group of rats used in the fentanyl studies (behavioral studies; blood pressure and heart rate studies; apnea studies and changes in body weight and body temperature studies) are provided in Supplemental Table 2. Note that there were no between group differences in body weights in any study (P > 0.05 for all comparisons). Also note that each rat was used in only one study protocol described below.

### Protocols to determine the abilities of L-NAC or L-NACme to prevent the development of physical dependence to fentanyl

#### Behavioral studies

Three groups of rats received a jugular vein catheter of PE-10 connected to PE-50 (*Intramedic; Becton & Dickinson, Franklin Drive, NJ, USA*) under 2–3% isoflurane anesthesia, to allow injections of test agents^36^. All rats received an intraperitoneal injection of the non-steroidal anti-inflammatory analgesic, carprofen (20 mg/kg) immediately post-surgery and again 24 later. Rats were given 4 days to recover from surgery. Study 1–5 co-injections: Groups of rats (n = 9 per group) received co-injections of (a) vehicle (100 μL/100g body weight, IV) + fentanyl (125 μg/kg, IV), (b) L-NAC (500 μmol/kg, IV) + fentanyl (125 μg/kg, IV), or (c) L-NACme (500 μmol/kg, IV) + fentanyl (125 μg/kg, IV) given 90 s apart at 8 AM and 8 PM on days 1 and 2 and at 8 AM on day 3. Study 1–10 co-injections: Groups of rats (n = 9 per group) received co-injections (a) vehicle (100 μL/100g body weight, IV) + fentanyl (125 μg/kg, IV), (b) L-NAC (500 μmol/kg, IV) + fentanyl (125 μg/kg, IV), or (c) L-NACme (500 μmol/kg, IV) + fentanyl (125 μg/kg, IV) given 90 s apart at 8 AM and 8 PM on days 1 to 4. The rats received injection 9 at 8 AM on day 5 and injection 10 at 2 PM to allow for subsequent NLX challenges to be given. Ninety min after the 5th or 10th set of co-injections, the rats were placed in individual opaque plastic boxes of 12″ in length by 6″ in width by 6″ in height, which allowed the rats to move around without constraint. After 30 min, they were injected with NLX (1.5 mg/kg, IV) and behavioral phenomena were scored for 45 min by 3 scorers who were blinded to any of the treatments that the rat had received. Scored phenomena were: Jumping behavior—all 4 paws of the ground—jumps; Wet dog shakes—whole body shakes as if to shed water from fur; Rearing behavior—rearing on hind legs—rears; Episodes of fore-paw licking – FPL; Circling behavior—Complete 360° rotation; Writhes – full body contortion; Sneezes—episodes of sneezing (abrupt expulsion of air that often disturbed the fine bedding material).

#### Plethysmography ventilatory studies

One hour before co-injections 5 or 10 were to be given (as described above), rats were placed into individual whole body plethysmography chambers to record ventilatory parameters^36^. The free end of the exteriorized venous catheter was connected to a swivel assembly housed in the lid of the plethysmography chamber and after 60 min acclimatization, the rats received co-injections 5 or 10 and after 90 min they received an injection of NLX (1.5 mg/kg, IV). The number of apneas of greater than 1.5 s in duration were determined by internal *FinePointe* software (DSI, Harvard Bioscience, Inc., St. Paul, MN)^36^.

#### Cardiovascular studies

Groups of rats (n = 9 rats per group) were implanted with a jugular vein catheter to inject drugs and a catheter into a femoral artery to continuously record mean arterial blood pressure (MAP) and heart rate^36^. The rats were given 5 days to recover from surgery. The arterial lines were kept patent throughout the acclimatization period and 3 or 5 day study protocols by connecting the arterial line to an infusion pump (*Standard Infuse-Withdraw Pump 11 Pico Plus Elite Programmable Syringe Pump; Harvard Apparatus, MA, USA*) delivering normal saline at 20 μL/h). One hour before co-injections 5 or 10 were to be given, the rats were placed in individual opaque plastic boxes and prepared for drug delivery to continuously record pulsatile arterial blood pressure to derive MAP  and heart rate. After 60 min acclimitization, the rats received co-injections 5 or 10, and after 90 min they received NLX (1.5 mg/kg, IV) and cardiovascular parameters recorded continuously for a further 90 min.

#### Body temperature and body weight studies

Groups of rats (n = 9 per group) were placed in individual opaque plastic boxes one hour before co-injections 5 or 10 of drug combinations were to be given (as above). A thermistor probe connected to a telethermometer (*Yellow Springs Instruments*) to record body temperature was inserted 5–6 cm into the rectum and taped to the tail^36^. Body weights of the rats and body temperatures were recorded every 15 min during acclimatization to establish baseline values and at 15 min intervals throughout the injection study protocols. After 60 min acclimatization, the rats received co-injections 5 or 10, and after 90 min they received an injection of NLX (1.5 mg/kg, IV) and body weights and body temperatures were recorded for a further 90 min.

### Protocols to determine the abilities of L-NAC or L-NACme to overcome fentanyl dependence

#### Behavioral studies

Groups of rats (n = 9 per group) received 5 injections of fentanyl (125 μg/kg, IV) at 8 AM and 8 PM as described above. These rats then received co-injections 6–10 of (a) fentanyl (125 μg/kg, IV) + vehicle, (b) L-NAC (500 μmol/kg, IV) + fentanyl (125 μg/kg, IV) or (c) L-NACme (500 μmol/kg, IV) + fentanyl (125 μg/kg, IV) given 90 s apart. Co-injections 6 were given at 8 PM, co-injections 7 at 8 AM, co-injections 8 at 8 PM, co-injections 9 at 8 AM, and co-injection 10 was given at 2 PM to allow for the experiments to be performed. Immediately after co-injections 10 were given, the rats were placed in individual opaque plastic boxes and after a 90 min period of acclimatization, the rats received an injection of NLX (1.5 mg/kg, IV) and behavioral phenomena (as detailed above) were scored for 45 min by at least 3 scorers.

#### Plethysmography ventilatory studies

One hour before co-injections 10 were to be given, rats were put into individual whole body plethysmography chambers to record ventilatory parameters. The free end of the exteriorized venous catheter was connected to the swivel assembly and after 60 min acclimatization, the rats received the 10th set of co-injections and after 90 min they received NLX (1.5 mg/kg, IV). Ventilatory parameters and non-eupneic breathing indices were recorded with the number of apneas of > 1.5 s in length reported here.

#### Cardiovascular studies

One hour before the 10th set of co-injections were given, rats (n = 9 rats per group) were put in individual opaque plastic boxes and the free end of the exteriorized jugular vein catheter was connected to an injection line to deliver drugs. The free end of the arterial catheter was connected to tubing attached to a computer-coupled pressure transducer (*Cabe Lab, Inc*.) to continuously record pulsatile arterial blood pressure to derive MAP and heart rate. After 60 min acclimatization, rats received co-injections 10 and then after 90 min an injection of NLX (1.5 mg/kg, IV) and cardiovascular parameters were recorded continuously for a further 90 min.

#### Body temperature and body weight studies

Groups of rats (n = 9 per group) were placed in individual opaque plastic boxes one hour before the 10th set of co-injections were to be given (as described above). A thermistor probe connected to a telethermometer (*Yellow Springs Instruments*) to record body temperature was inserted 5–6 cm into the rectum and taped to the tail^36^. Body weights of the rats and body temperatures were recorded every 15 min during acclimatization to establish baseline values and at 15 min intervals throughout the injection study protocols. After 60 min acclimatization, the rats received co-injections 10 and after 90 min they were injected with NLX (1.5 mg/kg, IV) and body weights and body temperatures were recorded for another 90 min.

#### Control studies

Rats (n = 9 per group) were used to determine the effects of NLX (1.5 mg/kg, IV) that had received co-injections of vehicle (saline) rather than the co-injections of fentanyl (Supplemental Table 2). In behavioral studies addressing whether NLX elicits behaviors, such as wet-dog shakes, rats received 5 or 10 co-injections of (a) vehicle + vehicle, (b) vehicle + L-NAC (500 μmol/kg, IV), or (c) vehicle + L-NACme (500 μmol/kg, IV). As above, these rats received the injection of NLX 90 min after the last set of co-injections. Other rats (n = 9 per group) described in Supplemental Table 2 were used to determine whether NLX (1.5 mg/kg, IV) elicited changes in body weights and body temperatures as described above.

### Data analyses

**Figure 1:**
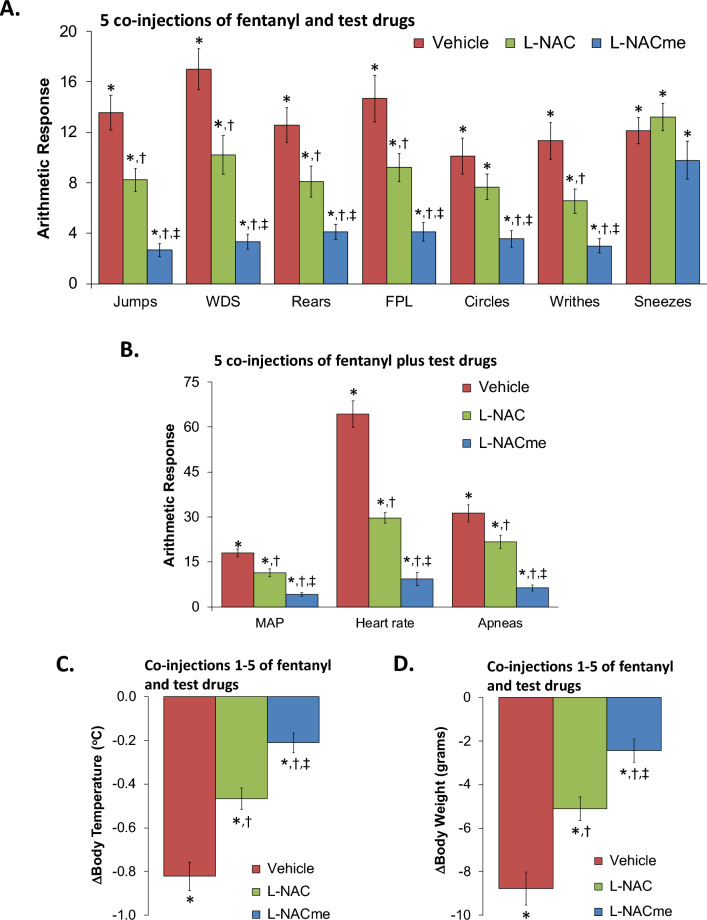
Responses elicited by injection of naloxone HCl (1.5 mg/kg, IV) in rats that had received 5 co-injections of fentanyl (125 μg/kg, IV) plus vehicle, N-acetyl-L-cysteine (L-NAC, 500 μmol/kg, IV) or L-NAC methyl ester (L-NACme, 500 μmol/kg, IV). (**A**) Behavioral responses – jumps, wet-dog shakes (WDS), rears, fore-paw licking (FPL), circles, writhes, and sneezes. (**B**) Cardiorespiratory responses, mean arterial blood pressure (MAP), heart rate and apneas. (**C**) Body temperatures. (**D**) Body weights. Data are shown as mean ± SEM. There were 9 rats in each group. *P < 0.05, significant response from pre-drug values. ^†^P < 0.05, L-NAC or L-NACme versus vehicle. ^‡^P < 0.05, L-NACme versus L-NAC.

**Figure 2:**
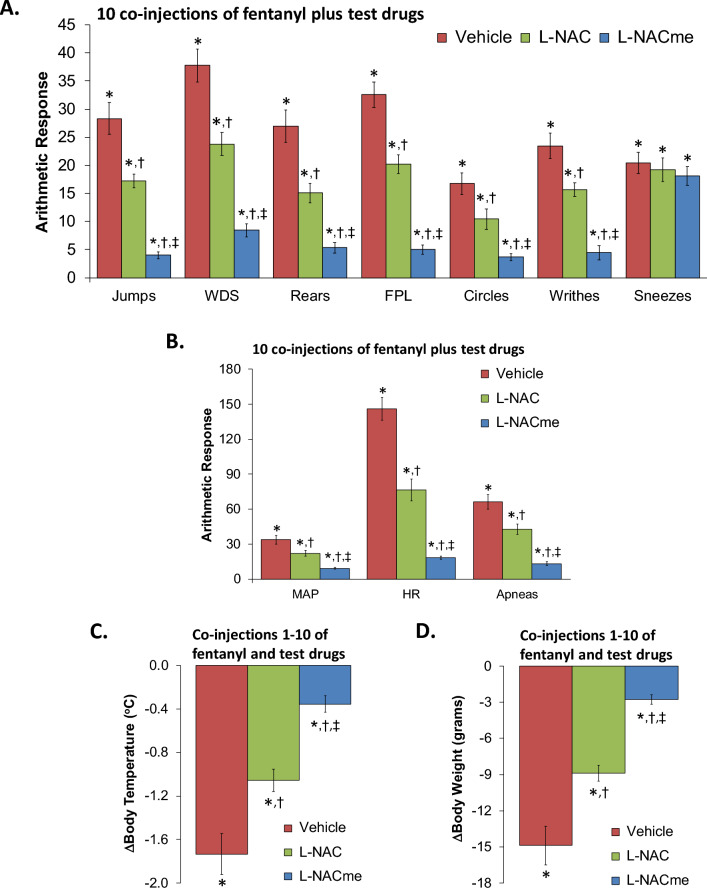
Responses elicited by injection of naloxone HCl (1.5 mg/kg, IV) in rats that had received 10 co-injections of fentanyl (125 μg/kg, IV) plus vehicle, N-acetyl-L-cysteine (L-NAC, 500 μmol/kg, IV) or L-NAC methyl ester (L-NACme, 500 μmol/kg, IV). (**A**) Behavioral responses – jumps, wet-dog shakes (WDS), rears, fore-paw licking (FPL), circles, writhes, and sneezes. (**B**) Cardiorespiratory responses, mean arterial blood pressure (MAP), heart rate and apneas. (**C**) Body temperatures. (**D**) Body weights. Data are shown as mean ± SEM. There were 9 rats in each group. *P < 0.05, significant response from pre-drug values. ^†^P < 0.05, L-NAC or L-NACme versus vehicle. ^‡^P < 0.05, L-NACme versus L-NAC.

**Figure 3:**
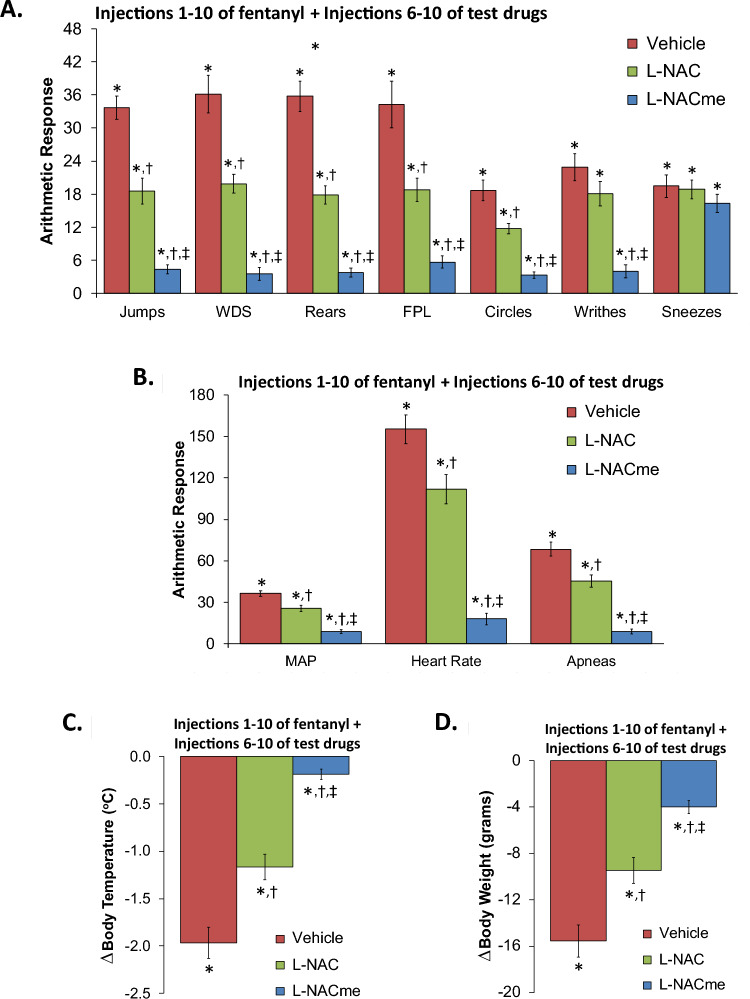
Responses elicited by injection of naloxone HCl (1.5 mg/kg, IV) in rats that had received 10 injections of fentanyl (125 μg/kg, IV) plus 5 co-injections of vehicle, N-acetyl-L-cysteine (L-NAC, 500 μmol/kg, IV) or L-NAC methyl ester (L-NACme, 500 μmol/kg, IV) beginning at fentanyl injection 6. (**A**) Behavioral responses – jumps, wet-dog shakes (WDS), rears, fore-paw licking (FPL), circles, writhes, and sneezes. (**B**) Cardiorespiratory responses, mean arterial blood pressure (MAP), heart rate and apneas. (**C**) Body temperatures. (**D**) Body weights. Data are shown as mean ± SEM. There were 9 rats in each group. *P < 0.05, significant response from pre-drug values. ^†^P < 0.05, L-NAC or L-NACme versus vehicle. ^‡^P < 0.05, L-NACme versus L-NAC.

All data are presented as mean ± SEM and were analyzed by one-way and two-way ANOVA followed by Bonferroni corrections for multiple comparisons between means using the error mean square terms from each ANOVA analysis as described previously^36^. A *P* < 0.05 value denoted the initial level of significance that was modified as per the number of comparisons between means. The modified *t-*statistic is t = (mean group 1–mean group 2)/[s × (1/n_1_ + 1/n_2_)^1/2^] where s^2^ = mean square within groups term from the ANOVA (the square root of this value is used in the modified *t*-statistic formula) and n_1_ and n_2_ are the number of rats in each group under comparison. Based on Bonferroni's inequality, a conservative critical value for modified *t*-statistics is obtained from tables of *t*-distribution using a significance level of P/m, where m is the number of comparisons between groups to be performed^36^. The degrees of freedom are those for the mean square for within group variation from the ANOVA table. In most situations, the critical Bonferroni value cannot be found in conventional tables of the *t*-distribution but can be approximated from tables of the normal curve by t* = z + (z + z^3^)/4n, with n being the degrees of freedom and z being the critical normal curve value for P/m^37^. Wallenstein et al.^37^ demonstrated that the Bonferroni procedure possesses the widest range of applications because it provides critical values that are lower than those of other procedures when the number of comparisons can be limited, and will be slightly larger than those of other procedures if many comparisons are made. Statistical analyses were performed with the aid of GraphPad Prism software (*GraphPad Software*, Inc., La Jolla, CA). F- and *P*-statistics associated with analyses of the data in Figs. 1, 2 and 3 are given in Supplemental Table 3.

### Ethics statement

The Institutional Animal Care and Use Committees (IACUC) of the University of Virginia, Case Western Reserve University and *Galleon Pharmaceuticals, Inc*. (Horsham, PA) provided official approval for the studies presented in this manuscript.

## Results

### NLX elicits negligible responses in rats that received co-injections of vehicle rather than fentanyl

As seen in Supplemental Table 4, the administration of NLX (1.5 mg/kg, IV) in the groups of rats that were co-injected with (a) vehicle + vehicle, (b) vehicle + L-NAC, or (c) vehicle + L-NACme (following the 5 or 10 co-injection protocols and following the protocol in which L-NAC or L-NACme were given beginning with co-injection 6 of vehicle) did not elicit any of the behaviors observed in rats that were co-injected with fentanyl. In addition, the injection of NLX did not elicit changes in body weights or body temperatures in rats that underwent the 5 or 10 co-injection protocols (Supplemental Table 5) or rats that underwent the protocol in which L-NAC and L-NACme were introduced with co-injection 6 of vehicle (Supplemental Table 6).

### L-NAC and L-NACme prevention of physical dependence to fentanyl

The behavioral phenomena elicited by the injection of NLX (1.5 mg/kg, IV) in rats that had received 5 co-injections of fentanyl (125 μg/kg, IV) + vehicle or L-NAC (500 μmol/kg, IV) or L-NACme (500 μmol/kg, IV) are shown in panel A of Fig. 1. Injection of NLX to rats that received fentanyl + vehicle elicited jumping behavior (jumps), wet-dog shakes (WDS), rearing behavior (rears), fore-paw licking (FPL), circling behavior (circles), full-body writhing (writhes), and episodes of sneezing (sneezes). NLX-precipitated phenomena (except for sneezing) were diminished in rats that received fentanyl + L-NAC, and more so in rats that received fentanyl + L-NACme. As seen in Panel B of Fig. 1, NLX (1.5 mg/kg)-induced increases in MAP, heart rate, and incidence of apneic events (> 1.5 s) were reduced by L-NAC, and more greatly reduced by L-NACme. As seen in Panels C and D of Fig. 1, the NLX-induced falls in body temperature and body weight, respectively, were less in rats that received fentanyl + L-NAC and even less in rats that received fentanyl + L-NACme. Behavioral phenomena elicited by the injection of NLX (1.5 mg/kg, IV) in rats that had received 10 co-injections of fentanyl (125 μg/kg, IV) + vehicle or L-NAC (500 μmol/kg, IV) or L-NACme (500 μmol/kg, IV) are shown in panel A of Fig. 2. The injection of NLX to rats that received fentanyl + vehicle elicited qualitatively similar responses as described above, except that they were more numerous. The NLX-precipitated withdrawal phenomena (except for the sneezing) were diminished in rats that had received fentanyl + L-NAC, and more greatly diminished in rats that had received fentanyl + L-NACme. As seen in Panel B of Fig. 2, the NLX (1.5 mg/kg)-induced increases in MAP, heart rate (HR), and incidence of apneic events (> 1.5 s) were reduced by L-NAC, and more diminished by L-NACme. As seen in Panels C and D of Fig. 2, the NLX-induced decreases in body weight and body temperature were diminished in rats that had received fentanyl + L-NAC and more diminished in rats that had received fentanyl + L-NACme.

### L-NAC and L-NACme reversal of established physical dependence to fentanyl

The behavioral responses elicited by the injection of NLX (1.5 mg/kg, IV) in rats that had received 10 injections of fentanyl (125 μg/kg, IV) plus 5 co-injections of fentanyl (125 μg/kg, IV) + vehicle, or L-NAC (500 μmol/kg, IV) or L-NACme (500 μmol/kg, IV) beginning at fentanyl injection 6 are summarized in Panel A of Fig. 3.

Sneezes aside, the NLX-precipitated phenomena were reduced in rats that received co-injections of L-NAC, and more reduced in rats that received L-NACme. As seen in Panel B of Fig. 3, the NLX-precipitated increases in MAP, heart rate, and incidence of apneas (> 1.5 s) were reduced in rats that received L-NAC, and more greatly reduced in those that received L-NACme. As seen in Panels C and D of Fig. 3, the NLX-induced decreases in body weight and body temperature, were not as decreased in rats that received fentanyl + L-NAC and were even less decreased in rats that received fentanyl + L-NACme.

### Changes in variables during the progression of the protocols

The actual body weights and arithmetic changes in weights at key points of Studies 1–4 for co-injections 1–5 and co-injections 1–10 studies are shown in Table 1. The rats that received fentanyl + vehicle lost body weight (arithmetic change from pre-drug values). In rats that received fentanyl + vehicle, the loss in body weight after 10 injections was greater than after 5 injections, whereas the increases in body weights in the rats that received fentanyl + L-NAC or fentanyl + L-NACme after 10 co-injections were greater than after 5 co-injections. Regarding the effects of NLX in co-injections 1–5 and 1–10 studies, the decreases in body weight were less in L-NAC-treated rats and even more less in L-NACme-treated rats. Actual body weights and arithmetic changes in weights at key points of Studies 1–4 for co-injections 6–10 studies are shown in Table 2. In rats that received fentanyl + vehicle, the loss in body weight after 10 injections were greater than after 5 injections, whereas the increases in body weights in the rats that received fentanyl + L-NAC or fentanyl + L-NACme after co-injections 10 were greater than after 5 co-injections. Regarding the effects of NLX, the decreases in body weight were less in L-NAC-treated rats and even more less in L-NACme-treated rats. Actual body temperatures and arithmetic changes in temperatures at key points of Studies 1–4 for co-injections 1–5 studies, co-injections 1–10 studies and co-injection 6–10 studies are shown in Table 3. The rats that received 5 or 10 co-injections of fentanyl + vehicle displayed a hyperthermia that was less pronounced in rats that received fentanyl + L-NAC and fentanyl + L-NACme. Similarly, the rats that received 10 injections of fentanyl plus co-injections of vehicle, L-NAC or L-NACme with injections 6–10 of fentanyl, displayed hyperthermia that was less intense in rats that received L-NAC and in those that received L-NACme. Again, the NLX-precipitated falls in body temperature seen in rats that received fentanyl + L-NAC and fentanyl + L-NACme were smaller than in fentanyl + vehicle rats.

**Table 1:** Actual body weights and their arithmetic changes at key points of study.

	Stage	Vehicle	L-NAC	L-NACme
Co-injection 1–5 studies				
Behavioral	Pre	336 ± 1	335 ± 1	336 ± 1
	Inj 5	329 ± 1	338 ± 1	342 ± 2
	Inj 5 vs pre	− 7.3 ± 1.4*	+ 3.6 ± 0.4*^,†^	+ 6.4 ± 1.0*^,†,‡^
MAP and heart rate	Pre	336 ± 2	336 ± 2	336 ± 1
	Inj 5	327 ± 1	340 ± 2	343 ± 2
	Inj 5 vs pre	− 8.3 ± 0.8*	+ 3.8 ± 0.3*^,†^	+ 6.9 ± 0.5*^,†,‡^
Apnea	Pre	335 ± 1	335 ± 1	337 ± 1
	Inj 5	327 ± 2	340 ± 2	344 ± 1
	Inj 5 vs pre	− 8.4 ± 0.9*	+ 4.7 ± 0.6*^,†^	+ 7.4 ± 0.5*^,†,‡^
BW and BT	Pre	336 ± 1	337 ± 1	337 ± 1
	Day 5	330 ± 1	340 ± 1	345 ± 1
	Post-NLX	321 ± 1	335 ± 1	342 ± 2
	Day 5 vs pre	− 6.7 ± 0.6*	+ 2.9 ± 0.4*^,†^	+ 8.1 ± 0.7*^,†,‡^
	Post-NLX vs day 5	− 8.8 ± 0.8*	− 5.1 ± 0.5*^,†^	− 2.4 ± 0.5*^,†,‡^
Co-injection 1–10 studies				
Behavioral	Pre	336 ± 1	336 ± 1	334 ± 1
	Day 10	324 ± 2	340 ± 2	344 ± 1
	Day 10 vs pre	− 12.0 ± 1.0*	+ 4.4 ± 0.6*^,†^	+ 10.0 ± 0.6*^,†,‡^
MAP and heart rate	Pre	335 ± 1	336 ± 2	335 ± 1
	Day 10	324 ± 2	341 ± 2	348 ± 1
	Day 10 vs pre	− 10.7 ± 0.9*	+ 5.1 ± 0.6*^,†^	+ 12.1 ± 0.8*^,†,‡^
Apnea	Pre	336 ± 2	336 ± 2	337 ± 1
	Day 10	325 ± 2	341 ± 2	349 ± 1
	Day 10 vs pre	− 11.3 ± 1.1*	+ 5.0 ± 0.6*^,†^	+ 11.3 ± 1.2*^,†,‡^
BW and BT	Pre	336 ± 1	335 ± 1	336 ± 1
	Day 10	325 ± 2	339 ± 1	348 ± 2
	Post-NLX	310 ± 3	310 ± 3	341 ± 2
	Day 10 vs pre	− 10.4 ± 1.1*	+ 4.1 ± 0.4*^,†^	+ 11.6 ± 1.4*^,†,‡^
	Post-NLX vs day 10	− 14.9 ± 1.6*	− 8.9 ± 0.7*^,†^	− 2.8 ± 0.4*^,†,‡^

*NLX* naloxone hydrochloride (1.5 mg/kg, IV), *MAP* mean arterial blood pressure, *L-NAC* N-acetyl-L-cysteine (500 μmol/kg, IV), *L-NACme* L-NAC methyl ester (500 μmol/kg, IV).

The data are shown as mean ± SEM. There were 9 rats in each group.

*P < 0.05, significant response from pre-drug values. ^†^P < 0.05, L-NAC or L-NACme versus vehicle. ^‡^P < 0.05, L-NACme versus L-NAC.

**Table 2:** Actual body weights and their arithmetic changes at key points of study.

Co-injection 6–10 studies	Stage	Vehicle	L-NAC	L-NACme
Behavioral	Pre	336 ± 1	336 ± 1	337 ± 1
	Pre-inj 6	328 ± 1	340 ± 1	343 ± 1
	Pre-inj 10	323 ± 1	342 ± 1	348 ± 2
	Pre-inj 6 versus pre	− 7.3 ± 0.9*	+ 3.8 ± 0.4*^,†^	+ 6.0 ± 0.6*^,†,‡^
	Pre-inj 10 versus pre	− 12.3 ± 1.1*	+ 5.9 ± 0.5*^,†^	+ 11.4 ± 0.9*^,†,‡^
	Pre-inj 10 versus pre-inj 6	− 5.0 ± 0.3*	+ 2.1 ± 0.4*^,†^	+ 5.4 ± 0.6*^,†,‡^
MAP, heart rate	Pre	336 ± 2	335 ± 1	337 ± 2
	Pre-inj 6	329 ± 1	339 ± 1	344 ± 1
	Pre-inj 10	323 ± 1	343 ± 1	350 ± 1
	Pre-inj 6 versus pre	− 7.6 ± 0.9*	+ 4.4 ± 0.6*^,†^	+ 6.9 ± 0.8*^,†^
	Pre-inj 10 versus pre	− 13.3 ± 1.3*	+ 8.1 ± 0.5*^,†^	+ 13.6 ± 1.0*^,†,‡^
	Pre-inj 10 versus pre-inj 6	− 5.8 ± 0.9*	+ 3.7 ± 0.4*^,†^	+ 6.7 ± 0.6*^,†,‡^
Apnea	Pre	335 ± 1	336 ± 2	338 ± 2
	Pre-inj 6	328 ± 1	340 ± 2	342 ± 1
	Pre-inj 10	324 ± 1	343 ± 2	349 ± 1
	Pre-inj 6 versus pre	− 6.4 ± 0.9*	+ 4.1 ± 0.5*^,†^	+ 8.3 ± 0.8*^,†,‡^
	Pre-inj 10 versus pre	− 11.1 ± 1.6*	+ 7.2 ± 0.6*^,†^	+ 15.8 ± 1.2*^,†,‡^
	Pre-inj 10 versus pre-inj 6	− 4.7 ± 1.0*	+ 3.1 ± 0.5*^,†^	+ 7.4 ± 0.8*^,†,‡^
BW and BT	Pre	335 ± 1	335 ± 2	335 ± 1
	Pre-inj 6	327 ± 2	340 ± 2	341 ± 1
	Pre-inj 10	322 ± 2	344 ± 2	349 ± 2
	Post-NLX	307 ± 2	335 ± 2	345 ± 2
	Pre-inj 6 versus pre	− 7.4 ± 0.6*	+ 4.6 ± 0.6*^,†^	+ 5.6 ± 0.6*^,†^
	Pre-inj 10 versus pre	− 12.2 ± 1.0*	+ 8.3 ± 0.7*^,†^	+ 13.7 ± 1.3*^,†,‡^
	Pre-inj 10 versus pre-inj 6	− 4.8 ± 0.5*	+ 3.8 ± 0.3*^,†^	+ 8.1 ± 1.6*^,†,‡^
	Post-NLX vs pre-inj 10	− 15.6 ± 1.4*	− 8.7 ± 0.9*^,†^	− 3.2 ± 10.5*^,†,‡^

The data are shown as mean ± SEM. There were 9 rats in each group.

*NLX* naloxone hydrochloride (1.5 mg/kg, IV), *MAP* mean arterial blood pressure, *L-NAC* N-acetyl-L-cysteine (500 μmol/kg, IV), *L*-*NACme* L-NAC methyl ester (500 μmol/kg, IV).

*P < 0.05, significant response from pre-drug values. ^†^P < 0.05, L-NAC or L-NACme versus vehicle. ^‡^P < 0.05, L-NACme versus L-NAC.

**Table 3:** Actual body temperatures and their arithmetic changes at key points of study.

Stage	Vehicle	L-NAC	L-NACme
Co-injection 1–5 studies			
Pre	37.4 ± 0.07	37.5 ± 0.06	37.5 ± 0.06
Post-Inj 5/pre-NLX	38.0 ± 0.10	37.8 ± 0.07	37.6 ± 0.07
Post-NLX	37.2 ± 0.12	37.4 ± 0.07	37.43 ± 0.09
Post-Inj 5 vs pre	+ 0.62 ± 0.05*	+ 0.38 ± 0.05*^,†^	+ 0.13 ± 0.04*^,†,‡^
Post-NLX vs post-Inj 5	– 0.82 ± 0.6*	– 0.47 ± 0.05*^,†^	+ 0.21 ± 0.05*^,†,‡^
Co-injection 1–10 studies			
Pre	37.6 ± 0.06	37.5 ± 0.05	37.5 ± 0.05
Post-Inj 10/pre-NLX	38.5 ± 0.08	38.0 ± 0.06	37.7 ± 0.06
Post-NLX	36.7 ± 0.17	36.9 ± 0.13	37.3 ± 0.07
Post-Inj 10 vs pre	+ 0.82 ± 0.05*	+ 0.51 ± 0.04*^,†^	+ 0.20 ± 0.03*^,†,‡^
Post-NLX vs post-Inj 10	– 1.73 ± 0.19*	– 1.06 ± 0.10*^,†^	– 0.36 ± 0.07*^,†,‡^
Co-injection 6–10 studies			
Pre	37.5 ± 0.06	37.5 ± 0.08	37.6 ± 0.09
Pre-inj 6	38.0 ± 0.10	37.9 ± 0.09	37.8 ± 0.09
Post Inj 10/pre-NLX	38.6 ± 0.11	38.2 ± 0.07	37.8 ± 0.08
Post-NLX	36.6 ± 0.14	37.1 ± 0.07	37.6 ± 0.08
Pre-inj 6 versus pre	+ 0.57 ± 0.06*	+ 0.31 ± 0.04*^,†^	+ 0.14 ± 0.03*^,†,‡^
Post-inj 10 versus pre	+ 1.10 ± 0.08*	+ 0.62 ± 0.05*^,†^	+ 0.18 ± 10.05*^,†,‡^
Post-inj 10 versus pre-inj 6	+ 0.53 ± 0.09*	+ 0.31 ± 0.08*^,†^	+ 0.03 ± 0.06*^,†,‡^
Post-NLX vs post-inj 10	− 1.96 ± 0.12*	− 1.03 ± 0.08*^,†^	– 0.19 ± 0.06*^,†,‡^

The data are shown as mean ± SEM. There were 9 rats in each group.

*NLX* naloxone hydrochloride (1.5 mg/kg, IV), *MAP* mean arterial blood pressure, *L*-*NAC* N-acetyl-L-cysteine (500 μmol/kg, IV), *L-NACme* L-NAC methyl ester (500 μmol/kg, IV).

*P < 0.05, significant response from pre-drug values. ^†^P < 0.05, L-NAC or L-NACme versus vehicle. ^‡^P < 0.05, L-NACme versus L-NAC.

The actual MAP, heart rate and Δheart rate/ΔMAP (cardiovascular index) values before and after administration of NLX, plus arithmetic changes in these parameters, for co-injections 1–5, co-injections 1–10 and co-injections 6–10 studies are shown in Table 4. There were no between-group differences in resting parameters prior to the administration of NLX (note that the injections of fentanyl elicited decreases in MAP and heart rate that had fully resolved by the time the pre-NLX measurements were taken. The development of tolerance to the cardiovascular effects of fentanyl will be reported elsewhere). The NLX-precipitated increases in MAP and heart rate in rats that received 10 co-injections of fentanyl + vehicle were greater than those that received 5 co-injections of fentanyl + vehicle. The NLX-precipitated increases in MAP and heart rate were smaller in rats that received fentanyl + L-NAC and fentanyl + L-NACme in the co-injections 1–5, co-injections 1–10 and co-injections 6–10 studies. Arithmetic changes in ΔHeart Rate/ΔMAP values (see column denoted Delta) were enhanced in the rats that received co-injections 1–10 and co-injections 6–10 of fentanyl + vehicle. These ratios were diminished in rats that received co-injections of fentanyl + L-NACme for the co-injections 1–5, co-injections 1–10 and co-injections 6–10 studies.

**Table 4:** Cardiorespiratory responses elicited by the injection of naloxone HCl.

Study	MAP (mmHg)			Heart rate (beats/min)			ΔHeart rate/ΔMAP (bpm/mmHg)		
	Pre	Post-NLX	Delta	Pre	Post-NLX	Delta	Pre	Post-NLX	Delta
Inj 1–5									
Vehicle	114 ± 2	132 ± 2	+ 18.0 ± 1.3*	359 ± 5	422 ± 7	+ 64.3 ± 4.4*	3.14 ± 0.05	3.20 ± 0.07	3.65 ± 0.07
L-NAC	114 ± 1	126 ± 2	+ 11.3 ± 1.3*	358 ± 6	387 ± 6	+ 29.7. ± 1.7*	3.13 ± 0.04	3.09 ± 0.06	2.83 ± 0.27
L-NACme	114 ± 1	118 ± 2	+ 4.2 ± 0.6*^,†^	356 ± 4	366 ± 4	+ 9.4 ± 2.2*^,†^	3.13 ± 0.04	3.10 ± 0.05	2.01 ± 0.26*^,†^
Inj 1–10									
Vehicle	114 ± 1	148 ± 4	+ 33.8 ± 3.6*	356 ± 4	501 ± 11	+ 145.8 ± 9.8*	3.11 ± 0.04	3.39 ± 0.04	4.48 ± 0.21*
L-NAC	113 ± 1	135 ± 3	+ 22.1 ± 2.4*	358 ± 6	434 ± 13	+ 76.2 ± 9.3*	3.17 ± 0.08	3.21 ± 0.09	3.44 ± 0.23*^,†^
L-NACme	114 ± 1	123 ± 1	+ 9.3 ± 0.7*^,†^	355 ± 5	373 ± 5	+ 18.4 ± 1.3*^,†^	3.11 ± 0.03	3.03 ± 0.03	2.01 ± 0.11*^,†^
Co-inj 6–10									
Vehicle	114 ± 1	151 ± 2	+ 36.4 ± 2.1*	355 ± 5	510 ± 13	+ 155.2 ± 10.4*	3.11 ± 0.06	3.39 ± 0.09	4.32 ± 0.32*
L-NAC	114 ± 1	140 ± 2	+ 25.4 ± 2.2*	354 ± 6	466 ± 9	+ 111.9 ± 10.7*	3.09 ± 0.07	3.34 ± 0.09	4.66 ± 0.57*
L-NACme	115 ± 1	124 ± 2	+ 8.9 ± 1.3*^,†^	356 ± 6	374 ± 6	+ 18.0 ± 4.1*^,†^	3.09 ± 0.05	3.02 ± 0.05	1.97 ± 0.21*^,†^

The data are shown as mean ± SEM. There were 9 rats in each group.

*MAP* mean arterial blood pressure, *Bpm* beats per minute, *NLX* naloxone hydrochloride (1.5 mg/kg, IV), *L-NAC* N-acetyl-L-cysteine (500 μmol/kg, IV), *L-NACme* L-NAC methyl ester (500 μmol/kg, IV).

*P < 0.05, significant response from pre-drug values. ^†^P < 0.05, L-NAC or L-NACme versus vehicle. ^‡^P < 0.05, L-NACme versus L-NAC.

## Discussion

The major objective driving the present study was to determine whether L-NAC and L-NACme are effective therapeutics to prevent and overcome physical dependence to fentanyl in rats. This study demonstrates that twice-daily injections of fentanyl (5 or 10 injections of 125 μg/kg, IV) elicit acute physical dependence in male Sprague Dawley rats on the basis of the pronounced withdrawal syndrome elicited by injection of NLX. The behavioral withdrawal signs, such as jumping, wet-dog shakes, rearing, circling, fore-paw licking, writhing, and sneezing, as well as the decreases in body weights and body temperatures, are consistent with the NLX-precipitated phenomena observed in fentanyl-administration protocols^35^ and in a variety of other opioid administration protocols used to induce dependence^36^. The increases in MAP and heart rate elicited by NLX are new findings with our fentanyl-dependence model, but are consistent with reports that NLX-precipitated withdrawal is associated with hypertension and tachycardia in experimental animals^36^ and humans^38^ due to globalized activation of sympathetic nerve activity. Additionally, our finding that NLX substantially increased apneic events (> 1.5 s) is a novel finding in our model, but also is consistent with previous findings in opioid withdrawal paradigms in rats and humans^36^. Moreover, our findings that NLX did not elicit behavioral signs or changes in body weights and body temperatures in rats that received co-injections of L-NAC or L-NACme with co- injections of vehicle rather than co-injections of fentanyl, suggests that neither L-NAC or L-NACme elicit acute physical dependence. These findings also suggest that the effects of L-NAC and L-NACme are due to interactions with the processes by which fentanyl elicits acute physical dependence.

Our first novel observations were that co-injections of L-NAC and L-NACme appeared to diminish the development of physical dependence to fentanyl on the basis that the NLX-precipitated withdrawal phenomena (e.g., behavioral responses, hypertension and tachycardia) were far less than in rats that received co-injections of fentanyl + vehicle. This may have been expected/predicted on the basis that co-injections of L-NAC and L-NACme reduced the body weight loss and development of hypothermia observed in the rats that received co-injections of fentanyl + vehicle. While the above findings are novel, Ward et al.^39^ found that acute systemic injection of L-NAC mitigated NLX-precipitated withdrawal in neonatal rats from mothers dependent on methadone, and prevented NLX-induced decreases in brain L-glutathione and L-glutamate levels. Our findings that L-NACme was much more effective than L-NAC in preventing the development of dependence to fentanyl is most likely due to it being more cell-penetrant than L-NAC^32^ and thus entering quickly into neurons of brain regions involved in the acquisition of physical dependence and addiction^40^.

The mechanisms by which L-NAC and L-NACme ameliorate the development of dependence to fentanyl may include (1) their antioxidant/reductant properties resulting in (a) modulation of redox status of molecules (e.g. reduction of L-cystine to L-cysteine), (b) alterations in activity of plasma membrane proteins, such as Kv_1.2_ K^+^-channels^41,^ and (c) redox modulation of intracellular proteins after entering into cells ^42^, (2) direct binding of L-NAC and L-NACme to plasma membrane/intracellular proteins, such as ion-channels, receptors and enzymes, that alters their activities by mechanisms not due to redox status of the proteins, (3) formation of thiol adducts, such as D-glucose:L-NAC akin to D-glucose:L-cysteine^43^ and disulfides of L-NAC and L-NACme^44^ and upon deacetylation to L-cysteine^45^, (4) formation of S-thiolated proteins, (5) generation of H_2_S, (6) conversion of L-thiolesters to cysteine sulfenics, and (7) formation of S-nitroso-L-cysteine^2^ with vital roles in cell signaling pathways including those in cardiorespiratory pathways^36^ and attenuation of OIRD^33,34^. These mechanisms may interact with brain pathways involved in acquisition of opioid dependence, such as those using N-methyl D-aspartate receptors^46^, muscarinic receptors^47^, corticotropin releasing factor (CRF) receptor CRF1^48^, tachykinin receptors^49^, voltage-gated Ca^2+^-channels^50^, opioid receptor phosphorylation^51^, oxidative stress^52^, and nitric oxide-cGMP cascades^53^. As L-NAC and L-NACme blunted all NLX-precipitated behavioral (except sneezing), physical (weight loss, hypothermia) and cardiorespiratory phenomena (hypertension, tachycardia, apneas), we assume that they interrupt key intracellular processes essential to development of opioid dependence. Interestingly, the ability of the redox regulator, α-lipoic acid, to diminish development of morphine dependence and to modulate NLX-induced biochemical alterations in morphine-dependent mice was enhanced by L-NAC^52^.

The second novel finding of this study was that the introduction of co-injections of L-NAC and L-NACme beginning with the 6th and continuing to the 10th injection of fentanyl, appeared to overcome established physical dependence to the opioid. More specifically, the NLX-precipitated behavioral phenomena (except sneezing), hypertension, tachycardia, apneic events, hypothermia and body weight loss were fewer in the rats that had received the co-injections of L-NAC and even fewer in those that received L-NACme. We do not know how L-NAC and L-NACme overcome physical dependence to fentanyl, but any/none of the mechanisms discussed above, including their potent antioxidant properties and abilities to boost intracellular levels of L-cysteine and L-glutathione, may be involved (see above). Agents that show efficacy at reversing established physical dependence include, L-histidine and histamine receptor sub-type agonists, melatonin, the antioxidant quercetin, the serotonin-reuptake inhibitor, fluoxetine, nitric oxide synthase inhibitors, Ca^2+^-calmodulin-dependent protein kinase II inhibitors, the β_2_-AR antagonist, butoxamine, adrenomedullin receptor antagonists, dopamine D2 receptor antagonists, ATP-dependent K^+^-channel modulators, and allosteric modulators of AMPA (α-amino-3-hydroxy-5-methyl-4-isoxazolepropionic acid) glutamate receptors^36,54^. The ability of L-NAC and L-NACme to overcome established physical dependence to fentanyl is of great clinical relevance, and opens the way for future studies on this and other bioactive L,D-thiol esters and related compounds^36^ to determine their the ability to overcome physical dependence to fentanyl and other opioids, such as heroin, and establishing pharmacological mechanisms of action.

A key question arising from these novel studies relates to the potential use of L- or D-thiolesters for key clinical problems associated with opioid analgesics. Regarding potential use in humans, (1) if L-NACme attenuates or blocks self-administration of opioids in OUD patients, adding it to prescription opioids may result in lower abuse or addiction potential; (2) if L-NACme attenuates or blocks development of physical dependence to opioids, then adding it to prescription opioids will minimize and may potentially eliminate physical dependence in individuals who receive opioids long-term (e.g., day in and day out for weeks or months); (3) if L-NACme attenuates or blocks tachyphylaxis to opioid analgesia or hyperalgesia caused by opioids in many people, then adding L-NACme to prescription opioids will maintain their analgesic efficacy over long periods of time, eliminating development of (a) tolerance, (b) need for escalating doses, and (c) potential complications of hyperalgesia; (4) if L-NACme has several of the advantageous effects found in rodents, then adding it to opioid analgesics would multiply beneficial aspects of opioids; (5) if L-NACme prevents the development of dependence, especially if introduced to an individual with physical dependence, and attenuates opioid withdrawal, it could be used as an outpatient/inpatient medication to manage opioid withdrawal in those iatrogenically physically-dependent (e.g., long-term opioid prescriptions) or those psychologically addicted and/or physically dependent; (6) if L-NACme attenuates or blocks euphoria and/or development of physiological dependence to opioids, then it would be a good medication for medication-assisted treatment (MAT) and a good drug for harm reduction interventions in people with OUD who are not interested in the psychosocial aspects of counseling and treatment; (7) as some patients with a history of OUD who are currently sober need opioids for treatment of acute or chronic pain syndromes, L-NACme, if it attenuates euphoria and physical dependence, could be added to opioid analgesics when given to people with a history of OUD, thereby eliminating the risk of opioid analgesics precipitating euphoria, drug cravings and their substantially increased risk of relapse; (8) if L-NACme attenuates or blocks euphoria from chemically-mediated dopamine surges in ventral tegmentum, nucleus accumbens or medial prefrontal cortex, where rewarding euphoria-producing dopamine surge happens from all drugs of abuse/addiction^40^, then it will be useful in treatment of OUD and other SUDs; and (9) if L-NACme attenuates or blocks euphoria from chemically-mediated dopamine surges, it could be combined with controlled prescription drugs resulting in an abuse-resistant or non-abusable form of prescribed opioids, for example.

A limitation of our study is that we have not examined whether lower doses of L-NAC and L-NACme prevent/overcome fentanyl-induced physical dependence. Establishing the lower limit is key to minimizing potential adverse biological effects that were not monitored in the present study. In addition, we do not know whether co-injections of L-NAC or L-NACme alter the antinociception actions of fentanyl (e.g., analgesia, expected tolerance, and occurrence of hyperalgesia), although we have reported that (a) bolus injections of L-NAC do not impair analgesia induced by continuous infusion of fentanyl in rats, despite reversing fentanyl-induced OIRD^20^, and (b) bolus injections of other L-D-thiolesters, such as D-cysteine di(m)ethyl ester, L- or D-cysteine (m)ethyl esters, and L-glutathione ethyl ester, prevent/overcome the actions of fentanyl and morphine on ventilatory parameters, and arterial blood-gas chemistry in rats without compromising opioid-induced analgesia or sedation^36^. Synthetic opioids, especially fentanyl, are playing a major and ever-increasing role in the current opioid crisis^55^ and future studies must determine whether L-NACme can overcome physical dependence to fentanyl in humans. Another vital limitation of our studies is the lack of data about the efficacy of L-NAC and L-NACme in preventing and reversing dependence to fentanyl in female rats. This is especially because opioids exert qualitatively and quantitatively different responses (e.g. ventilation, analgesia) in females than in males, such as sex-specific differences in opioid receptor signaling, sex differences in the development of opioid hyperalgesia, tolerance and withdrawal, and sex differences in expression and treatment of OUD^56,57^. The lack of understanding about the molecular mechanisms by which L-NAC and L-NACme affect the acquisition or overcome fentanyl dependence is another limitation that needs to be addressed. In addition to potential direct interactions with functional proteins, potential mechanisms of action of L-NACme may involve (1) direct binding to L,D-cysteine binding protein myristoylated alanine-rich C-kinase substrate, (2) interruption of μ-OR-β-arrestin-coupled cell signaling processes to spare the antinociceptive G-protein-dependent actions of morphine (and presumably fentanyl), and/or (3) potential conversion of L-NACme to S-nitroso-L-NACme, which has unique pharmacological profiles for oral use as a supplier of nitric oxide, L-glutathione and H_2_S, which may act in a similar way to the intracellular penetrating S-nitroso-L-cysteine ethyl ester^20^. Finally, a major limitation is our lack of information about distribution of L-NAC and L-NACme in brain regions relevant to acquisition of opioid dependence. We intend to perform pharmacokinetics analyses of L-NACme distribution using established liquid chromatography-mass spectrometry methodologies.

In conclusion, this study demonstrates that systemic injection of L-NAC and especially the membrane-permeable L-thiol ester, L-NACme, prevents the development of physical dependence to fentanyl and overcomes established dependence to fentanyl in male Sprague Dawley rats. Delineating the exact thiol-dependent signaling pathways by which L-NAC and L-NACme exert their effects will add to our understanding of the processes by which opioid induce dependence and how bioactive L-thiol esters exert their effects. Our study was spurred by the ground-breaking work of Trivedi, Deth and colleagues which added greatly to our understanding of the mechanisms by which opioids cause physical dependence and psychological addiction^13,27^. Their evidence that morphine may cause dependence/addiction by blocking the entry of L-cysteine into neurons by inhibition of EAA3/EAAC1 transporter^27^, prompted our studies with the membrane-permeable, L-cysteine ethyl ester (findings to be submitted), L-NAC (because of immediate clinical applicability) and L-NACme. Findings that L-NACme reduced the large majority of NLX-precipitated phenomena speaks to the key role that the loss of L-cysteine entry into cells has in physical dependence to fentanyl. The one withdrawal event not ameliorated by L-NACme was sneezing, a key feature of the opioid withdrawal response in humans and experimental animals^36^. We are trying to develop an understanding of the neural mechanisms responsible for sneezing to gain insights into the signaling pathways involved in the actions of L-NACme.

As to contributions to the field, this study provides a strong rationale for consideration of L-NACme and other bioactive membrane-permeable L-thiolesters, such as such as L-cysteine (m)ethyl ester, L-glutathione ethyl ester, γ-L-glutamylcysteine ethyl ester and L-cystine di(m)ethylester (see Supplemental Table 2 of Getsy et al.^58^) as therapeutics to prevent/overcome physical dependence to opioids. The recent report that L-NAC partially mitigates opioid withdrawal behaviors in neonatal rats via the reduction in brain oxidative stress^39^, raises the intriguing possibility that L-NACme or L-NAC ethyl ester, which is a more efficacious antioxidant than L-NAC^31^, will have greater efficacy in neonates and prove to be an effective therapeutic in male and female adults with OUD. Moreover, ethyl amide derivatives, such as the potent antioxidant, L-NAC ethyl amide^59^, may have greater efficacy against fentanyl than L-NAC (m)ethyl esters based on greater resistance to desterfication by carboxylesterases^60^. Furthermore, maternal opioid use is a growing public health concern and infants born from opioid-dependent mothers exhibit a variety of withdrawal symptoms often requiring weeks of hospitalization^61^. Current treatment strategies of neonatal opioid withdrawal syndrome (NOWS) are far from ideal and the infants go on to develop behavioral, social and cognitive deficits later in life^62^. More refined/alternative NOWS treatment strategies are needed as is a better understanding of the opioids mechanistic/molecular bases to drive optimization of effective therapeutic interventions. Given our compelling findings in adult male rats, further studies, including pharmacokinetic/tissue distribution and toxicology studies, are necessary to determine the potential therapeutic benefit of L-NAC and its more cell-penetrant analogues in treating NOWS in male and female rats. Moreover, since L-NAC is licensed for chronic use in humans, it would appear that a clinical trial with L-NAC in male and female subjects with fentanyl use-disorder could be performed in the near future.

### Supplementary Information

Supplementary Information.

## Data Availability

The author Stephen J. Lewis will provide the datasets generated from this study upon direct email request to sjl78@case.edu.

## Abbreviations

AMPA: α-Amino-3-hydroxy-5-methyl-4-isoxazolepropionic acid
CRF: Corticotropin releasing factor
EAA3: Excitatory amino acid transporter
FPL: Fore-paw licking
IV: Intravenous
L-NAC: N-acetyl-L-cysteine
L-NACme: N-acetyl-L-cysteine methyl ester
Kv_1.2_ K^+^-channels: Voltage-gated K^+^_1.2_ channels
μ-OR: μ-Opioid receptor
MAP: Mean arterial blood pressure
NLX: Naloxone hydrochloride
NOWS: Neonatal opioid withdrawal syndrome
OIRD: Opioid-induced respiratory depression
OUD: Opioid use disorder
SAM: S-adenosyl-methionine
SAH: S-adenosyl-homocysteine
SUD: Substance use disorder
WDS: Wet dog shakes
