# Supplementary material for: L-NAC and L-NAC methyl ester prevent and overcome physical dependence to fentanyl in male rats
Source: Sci Rep. 2024 Apr 20;14:9091. doi: 10.1038/s41598-024-59551-0 (PMC11032344; doi:10.1038/s41598-024-59551-0)
Supplement: Supplementary file 1 — Supplementary Information. [file 41598_2024_59551_MOESM1_ESM.docx]

**Supplemental File**

**L-NAC and L-NAC methyl ester prevent and reverse physical dependence**

**to fentanyl in male rats**

James N. Bates,^1,†^ Santhosh M. Baby,^2,‡^ Paulina M. Getsy,^3^ Gregory A. Coffee,^3^

Yee-Hsee Hsieh,^4^ Zackery T. Knauss,^5^ Albert Dahan,^6^ Jason A. Bubier,^7^

Peter M. MacFarlane,^3^ Devin Mueller,^5.^ Stephen J. Lewis^3,8,9,^*

*^1^Department of Anesthesiology, University of Iowa Hospitals and Clinics, Iowa City, Iowa, USA*

*^2^Section of Biology, Galleon Pharmaceuticals, Inc, Horsham, Pennsylvania, USA*

*^3^Department of Pediatrics, Division of Pulmonology, Allergy, and Immunology, Case Western Reserve University, Cleveland, Ohio, USA*

*^4^Division of Pulmonary, Critical Care and Sleep Medicine, Case Western Reserve University,*

*Cleveland, Ohio, USA*

*^5^Department of Biological Sciences, Kent State University, Kent, Ohio, USA*

*^6^Dept of Anesthesiology, Leiden University Medical Center, Leiden, the Netherlands*

*^7^Jackson Laboratories, Bar Harbor, Maine, USA*

*^8^Department of Pharmacology,* *Case Western Reserve University, Cleveland, Ohio, USA*

*^9^Functional Electrical Stimulation Center, Case Western Reserve University, Cleveland, Ohio, USA*

**^†^Present Address:** James N. Bates, Chief Medical Officer, *Atelerix Life Sciences Inc*. Address: 300 East Main Street, Suite 202 Charlottesville, Virginia 22902. Email: jbates@atelerixlifesciences.com. https://atelerixlifesciences.com

**^‡^Present address:** Translational Sciences Treatment Discovery, Galvani Bioelectronics, Inc, 1250 S Collegeville Rd, Collegeville, PA 1r9426, USA. Email: babysanthosh@gmail.com

***Corresponding Author:** Stephen J. Lewis, PhD. Department of Pediatrics, Division of Pulmonology, Allergy and Immunology, School of Medicine, Case Western Reserve University, 10900 Euclid Avenue, Cleveland, OH 44106-4984. Email: sjl78@case.edu

**Structures of L-NAC and analogues**

**L-NACme**

**L-NAC**


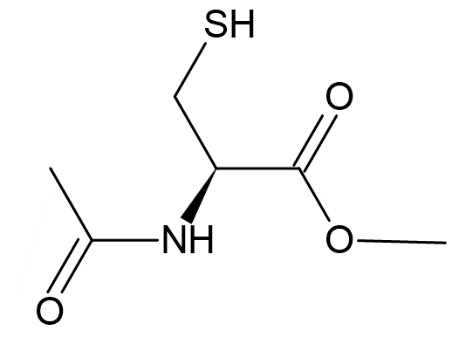

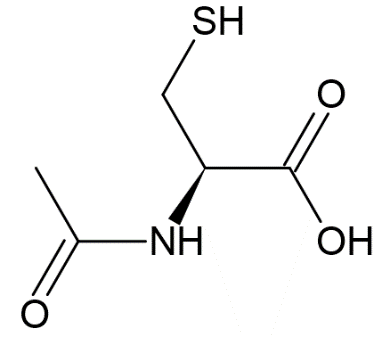


***N-acetyl-L-cysteine methyl ester***

*methyl (2R)-2-acetamido-3-sulfanylpropanoate*

***N-acetyl-L-cysteine***

*(2R)-2-acetamido-3-sulfanylpropanoic acid*

**L-NACma**

**L-NACpe**


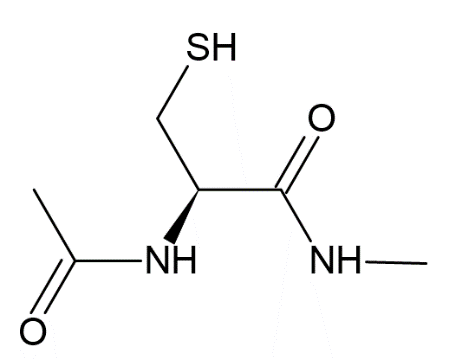

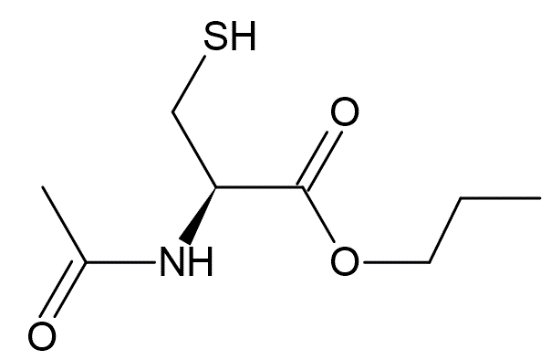


***N-acetyl-L-cysteine methyl amide***

*methyl (2R)-2-acetamido-3-sulfanylpropanamide*

***N-acetyl-L-cysteine propyl ester***

*propyl (2R)-2-acetamido-3-sulfanylpropanoate*

**Supplemental Figure 1.** Chemical structures of L-NAC and analogues.

**Supplemental Table 1.** Citations on the use of N-acetyl-L-cysteine in substance use disorders

**Alcohol**

Ferreira Seiva FR, Amauchi JF, Ribeiro Rocha KK, Souza GA, Ebaid GX, Burneiko RM, Novelli EL (2009) Effects of N-acetylcysteine on alcohol abstinence and alcohol-induced adverse effects in rats. *Alcohol* 43, 127-135. doi: 10.1016/j.alcohol.2008.12.003.

Schneider R Jr, Santos CF, Clarimundo V, Dalmaz C, Elisabetsky E, Gomez R (2015) N-acetylcysteine prevents behavioral and biochemical changes induced by alcohol cessation in rats. *Alcohol* 49, 259-263. doi: 10.1016/j.alcohol.2015.01.009.

Yawalkar R, Changotra H, Gupta GL (2018) Protective influences of N-acetylcysteine against alcohol abstinence-induced depression by regulating biochemical and GRIN2A, GRIN2B gene expression of NMDA receptor signaling pathway in rats. *Neurochem Int*. 118, 73-81. doi: 10.1016/j.neuint.2018.04.011.

Cano-Cebrián MJ, Fernández-Rodríguez S, Hipólito L, Granero L, Polache A, Zornoza T (2021) Efficacy of N-acetylcysteine in the prevention of alcohol relapse-like drinking: Study in long-term ethanol-experienced male rats. *J Neurosci Res*. 99, 638-648. doi: 10.1002/jnr.24736.

Fernández-Rodríguez S, Cano-Cebrián MJ, Esposito-Zapero C, Pérez S, Guerri C, Zornoza T, Polache A (2023) N-Acetylcysteine normalizes brain oxidative stress and neuroinflammation observed after protracted ethanol abstinence: a preclinical study in long-term ethanol-experienced male rats. *Psychopharmacology (Berl)*. 240, 725-738. doi: 10.1007/s00213-023-06311-z.

**Cannabis**

Marshall K, Gowing L, Ali R, Le Foll B (2014) Pharmacotherapies for cannabis dependence. *Cochrane Database Syst Rev*. 12, CD008940. doi: 10.1002/14651858.CD008940.pub2.

Sherman BJ, McRae-Clark AL, Baker NL, Sonne SC, Killeen TK, Cloud K, Gray KM (2017) Gender differences among treatment-seeking adults with cannabis use disorder: Clinical profiles of women and men enrolled in the achieving cannabis cessation-evaluating N-acetylcysteine treatment (ACCENT) study. *Am J Addict*. 26, 136-144. doi: 10.1111/ajad.12503.

Nielsen S, Gowing L, Sabioni P, Le Foll B (2019) Pharmacotherapies for cannabis dependence. *Cochrane Database Syst Rev*. 1, CD008940. doi: 10.1002/14651858.CD008940.pub3.

Tomko RL, Baker NL, Hood CO, Gilmore AK, McClure EA, Squeglia LM, McRae-Clark AL, Sonne SC, Gray KM (2020) Depressive symptoms and cannabis use in a placebo-controlled trial of N-Acetylcysteine for adult cannabis use disorder. *Psychopharmacology (Berl)*. 237, 479-490. doi: 10.1007/s00213-019-05384-z.

Sharma R, Tikka SK, Bhute AR, Bastia BK (2022) N-acetyl cysteine in the treatment of cannabis use disorder: A systematic review of clinical trials. *Addict Behav*. 129, 107283. doi: 10.1016/j.addbeh.2022.107283.

**Cocaine**

Baker DA, McFarland K, Lake RW, Shen H, Toda S, Kalivas PW (2003) N-acetyl cysteine-induced blockade of cocaine-induced reinstatement. *Ann N Y Acad Sci*. 1003, 349-351. doi: 10.1196/annals.1300.023.

LaRowe SD, Mardikian P, Malcolm R, Myrick H, Kalivas P, McFarland K, Saladin M, McRae A, Brady K (2006) Safety and tolerability of N-acetylcysteine in cocaine-dependent individuals. *Am J Addict*. 15, 105-110. doi: 10.1080/10550490500419169.

Amen SL, Piacentine LB, Ahmad ME, Li SJ, Mantsch JR, Risinger RC, Baker DA (2011) Repeated N-acetyl cysteine reduces cocaine seeking in rodents and craving in cocaine-dependent humans. *Neuropsychopharmacology* 36, 871-878. doi: 10.1038/npp.2010.226.

Reichel CM, Moussawi K, Do PH, Kalivas PW, See RE (2011) Chronic N-acetylcysteine during abstinence or extinction after cocaine self-administration produces enduring reductions in drug seeking. *J Pharmacol Exp Ther*. 337, 487-93. doi: 10.1124/jpet.111.179317.

Nocito Echevarria MA, Andrade Reis T, Ruffo Capatti G, Siciliano Soares V, da Silveira DX, Fidalgo TM (2017) N-acetylcysteine for treating cocaine addiction - A systematic review. *Psychiatry Res*. 251, 197-203. doi: 10.1016/j.psychres.2017.02.024.

**Methamphetamine**

Mousavi SG, Sharbafchi MR, Salehi M, Peykanpour M, Karimian Sichani N, Maracy M (2015) The efficacy of N-acetylcysteine in the treatment of methamphetamine dependence: a double-blind controlled, crossover study. *Arch Iran Med*. 18, 28-33.

McKetin R, Dean OM, Baker AL, Carter G, Turner A, Kelly PJ, Berk M (2017) A potential role for N-acetylcysteine in the management of methamphetamine dependence. *Drug Alcohol Rev*. 36, 153-159. doi: 10.1111/dar.12414.

Swanepoel T, Möller M, Harvey BH (2018) N-acetyl cysteine reverses bio-behavioural changes induced by prenatal inflammation, adolescent methamphetamine exposure and combined challenges. *Psychopharmacology (Berl).* 235, 351-368. doi: 10.1007/s00213-017-4776-5.

McKetin R, Dean OM, Turner A, Kelly PJ, Quinn B, Lubman DI, Dietze P, Carter G, Higgs P, Baker AL, Sinclair B, Reid D, Manning V, Te Pas N, Liang W, Thomas T, Bathish R, Kent M, Raftery D, Arunogiri S, Cordaro F, Hill H, Berk M (2019) A study protocol for the N-ICE trial: A randomised double-blind placebo-controlled study of the safety and efficacy of N-acetyl-cysteine (NAC) as a pharmacotherapy for methamphetamine ("ice") dependence. *Trials* 20, 325. doi: 10.1186/s13063-019-3450-0.

McKetin R, Dean OM, Turner A, Kelly PJ, Quinn B, Lubman DI, Dietze P, Carter G, Higgs P, Sinclair B, Reid D, Baker AL, Manning V, Pas NT, Thomas T, Bathish R, Raftery DK, Wrobel A, Saunders L, Arunogiri S, Cordaro F, Hill H, Hall S, Clare PJ, Mohebbi M, Berk M (2021) N-acetylcysteine (NAC) for methamphetamine dependence: A randomised controlled trial. *EClinicalMedicine* 38, 101005. doi: 10.1016/j.eclinm.2021.101005.

**Nicotine**

Schmaal L, Berk L, Hulstijn KP, Cousijn J, Wiers RW, van den Brink W (2011) Efficacy of N-acetylcysteine in the treatment of nicotine dependence: a double-blind placebo-controlled pilot study. *Eur Addict Res*. 17, 211-216. doi: 10.1159/000327682.

Froeliger B, McConnell PA, Stankeviciute N, McClure EA, Kalivas PW, Gray KM (2015) The effects of N-Acetylcysteine on frontostriatal resting-state functional connectivity, withdrawal symptoms and smoking abstinence: A double-blind, placebo-controlled fMRI pilot study. *Drug Alcohol Depend*. 156, 234-242. doi: 10.1016/j.drugalcdep.2015.09.021.

Bowers MS, Jackson A, Maldoon PP, Damaj MI (2016) N-acetylcysteine decreased nicotine reward-like properties and withdrawal in mice. *Psychopharmacology (Berl)*. 233, 995-1003. doi: 10.1007/s00213-015-4179-4.

Schulte M, Goudriaan AE, Kaag AM, Kooi DP, van den Brink W, Wiers RW, Schmaal L (2017) The effect of N-acetylcysteine on brain glutamate and gamma-aminobutyric acid concentrations and on smoking cessation: A randomized, double-blind, placebo-controlled trial. *J Psychopharmacol*. 31, 1377-1379. doi: 10.1177/0269881117730660.

Mocelin R, Marcon M, da Rosa Araujo AS, Herrmann AP, Piato A (2019) Withdrawal effects following repeated ethanol exposure are prevented by N-acetylcysteine in zebrafish. Prog *Neuropsychopharmacol Biol Psychiatry* 93, 161-170. doi: 10.1016/j.pnpbp.2019.03.014.

Nall RW, Beloate LN, Meyerink ME, Penaloza T, Doolittle J, Froeliger B, Kalivas PW, Garcia-Keller C (2022) Assessing combined effects of varenicline and N-acetylcysteine on reducing nicotine seeking in rats. *Addict Biol*. 27, e13151. doi: 10.1111/adb.13151.

**Supplemental Table 2**

Body weights for each group of rats used in the described studies

|  |  |  |  |  |  | **Treatment groups** | | | | |
| --- | --- | --- | --- | --- | --- | --- | --- | --- | --- | --- |
| **Study** |  | **Test** |  | **Protocol** |  | **Vehicle** |  | **L-NAC** |  | **L-NACme** |
| **A. Fentanyl groups** |  | Behaviors |  | Inj1-5 |  | 336 ± 1.2 |  | 335 ± 1.1 |  | 336 ± 1.3 |
| 36 groups, 9 rats per group |  |  |  | Inj1-10 |  | 336 ± 1.4 |  | 336 ± 1.3 |  | 334 ± 1.2 |
| 324 rats in total |  |  |  | Inj6-10 |  | 335 ± 1.2 |  | 336 ± 1.4 |  | 337 ± 1.2 |
|  |  | MAP, HR |  | Inj1-5 |  | 336 ± 1.6 |  | 336 ± 1.8 |  | 336 ± 1.3 |
|  |  |  |  | Inj1-10 |  | 335 ± 1.0 |  | 336 ± 1.7 |  | 335 ± 1.2 |
|  |  |  |  | Inj6-10 |  | 336 ± 1.5 |  | 335 ± 1.3 |  | 337 ± 1.6 |
|  |  | Apneas |  | Inj1-5 |  | 335 ± 1.2 |  | 335 ± 1.4 |  | 337 ± 1.1 |
|  |  |  |  | Inj1-10 |  | 336 ± 1.6 |  | 336 ± 1.7 |  | 337 ± 1.4 |
|  |  |  |  | Inj6-10 |  | 335 ± 1.3 |  | 336 ± 1.6 |  | 334 ± 1.5 |
|  |  | BW, BT |  | Inj1-5 |  | 336 ± 1.1 |  | 337 ± 1.2 |  | 337 ± 1.1 |
|  |  |  |  | Inj1-10 |  | 336 ± 0.8 |  | 335 ± 0.8 |  | 336 ± 1.4 |
|  |  |  |  | Inj6-10 |  | 335 ± 1.2 |  | 335 ± 1.5 |  | 336 ± 1.3 |
| **B. Vehicle groups** |  | Behaviors |  | Inj1-5 |  | 337 ± 1.3 |  | 335 ± 1.5 |  | 336 ± 1.5 |
| 18 groups |  |  |  | Inj1-10 |  | 334 ± 1.5 |  | 337 ± 1.7 |  | 337 ± 1.5 |
| 9 rats per group |  |  |  | Inj6-10 |  | 337 ± 1.5 |  | 335 ± 1.6 |  | 337 ± 1.4 |
| 162 rats in total |  | BW, BT |  | Inj1-5 |  | 336 ± 1.1 |  | 337 ± 1.2 |  | 335 ± 1.4 |
|  |  |  |  | Inj1-10 |  | 335 ± 1.3 |  | 337 ± 1.2 |  | 336 ± 1.2 |
|  |  |  |  | Inj6-10 |  | 336 ± 1.0 |  | 337 ± 1.4 |  | 336 ± 1.3 |

L-NAC, N-acetyl-L-cysteine (500 μmol/kg, IV); L-NACme, L-NAC methyl ester (500 μmol/kg, IV). NLX, naloxone hydrochloride (1.5 mg/kg, IV). MAP, mean arterial blood pressure. HR, heart rate. BW, body weight. BT, body temperature. The data are shown as mean ± SEM. There were 9 rats in each group. There were no between group differences for any body weight value (P > 0.05, for all comparisons).

**Supplemental Table 3.**

ANOVA descriptors

**Figure 1**

**Panel A:** Jumps (F_2,24_ = 29.14, P < 0.0001); WDS (F_2,24_ = 26.43, P < 0.0001); Rears (F_2,24_ = 14.27, P < 0.0001); FPL (F_2,24_ = 16.25, P < 0.0001); Circles (F_2,24_ = 9.52, P = 0.0009); Writhes (F_2,24_ = 15.59, P < 0.0001); Sneezes (F_2,24_ = 2.07, P = 0.15)**. Panel B:** MAP (F_2,24_ = 38.04, P < 0.0001); Heart rate (F_2,24_ = 86.50, P < 0.0001); Apneas (F_2,24_ = 64.37, P < 0.0001). **Panel C:** Body temperature (F_2,24_ = 26.42, P < 0.0001). **Panel D:** Body weights (F_2,24_ = 32.58, P < 0.0001).

**Figure 2: Panel A:** Jumps (F_2,24_ = 45.43, P < 0.0001); WDS (F_2,24_ = 45.01, P < 0.0001); Rears (F_2,24_ = 29.40, P < 0.0001); FPL (F_2,24_ = 66.19, P < 0.0001); Circles (F_2,24_ = 17.20, P = 0.0009); Writhes (F_2,24_ = 33.04, P < 0.0001); Sneezes (F_2,24_ = 0.37, P = 0.70)**. Panel B:** MAP (F_2,24_ = 23.42, P < 0.0001); Heart rate (F_2,24_ = 66.66, P < 0.0001); Apneas (F_2,24_ = 34.63, P < 0.0001). **Panel C:** Body temperature (F_2,24_ = 27.98, P < 0.0001). **Panel D:** Body weights (F_2,24_ = 34.28, P < 0.0001).

**Figure 3: Panel A:** Jumps (F_2,24_ = 62.28, P < 0.0001); WDS (F_2,24_ = 50.03, P < 0.0001); Rears (F_2,24_ = 71.22, P < 0.0001); FPL (F_2,24_ = 25.85, P < 0.0001); Circles (F_2,24_ = 38.19, P = 0.0009); Writhes (F_2,24_ = 23.52, P < 0.0001); Sneezes (F_2,24_ = 0.83, P = 0.45)**. Panel B:** MAP (F_2,24_ = 53.19, P < 0.0001); Heart rate (F_2,24_ = 61.65, P < 0.0001); Apneas (F_2,24_ = 53.57, P < 0.0001). **Panel C:** Body temperature (F_2,24_ = 95.22, P < 0.0001). **Panel D:** Body weights (F_2,24_ = 38.60, P < 0.0001).

**Supplemental Table 4**

Behavioral responses elicited by NLX in rats that received multiple co-injections of vehicle + vehicle, vehicle + L-NAC or vehicle + L-NACme

|  |  |  |  | **NLX-precipitated withdrawal behaviors** | | | | | | | | | | | | |
| --- | --- | --- | --- | --- | --- | --- | --- | --- | --- | --- | --- | --- | --- | --- | --- | --- |
| **Injections** |  | **Agent** |  | **Jumps** |  | **WDS** |  | **Rears** |  | **FPL** |  | **Circles** |  | **Sneezes** |  | **Writhes** |
| **Inj1-5** |  | Vehicle |  | 0.9 ± 0.4 |  | 0.7 ± 0.3 |  | 0.7 ± 0.4 |  | 0.8 ± 0.4 |  | 0.2 ± 0.2 |  | 0.7 ± 0.4 |  | 0.3 ± 0.2 |
|  |  | L-NAC |  | 0.1 ± 0.1 |  | 0.1 ± 0.1 |  | 0.2 ± 0.2 |  | 0.2 ± 0.2 |  | 0.1 ± 0.1 |  | 0.2 ± 0.2 |  | 0.0 ± 0.0 |
|  |  | L-NACme |  | 0.0 ± 0.0 |  | 0.0 ± 0.0 |  | 0.3 ± 0.3 |  | 0.2 ± 0.1 |  | 0.0 ± 0.0 |  | 0.0 ± 0.0 |  | 0.0 ± 0.0 |
| **Inj1-10** |  | Vehicle |  | 0.2 ± 0.1 |  | 0.7 ± 0.3 |  | 0.4 ± 0.3 |  | 1.0 ± 0.4 |  | 0.3 ± 0.2 |  | 0.8 ± 0.4 |  | 0.3 ± 0.2 |
|  |  | L-NAC |  | 0.1 ± 0.1 |  | 0.0 ± 0.0 |  | 0.2 ± 0.2 |  | 0.3 ± 0.3 |  | 0.1 ± 0.1 |  | 0.6 ± 0.3 |  | 0.0 ± 0.0 |
|  |  | L-NACme |  | 0.0 ± 0.0 |  | 0.0 ± 0.0 |  | 0.1 ± 0.1 |  | 0.3 ± 0.2 |  | 0.0 ± 0.0 |  | 0.0 ± 0.0 |  | 0.0 ± 0.0 |
| **Inj6-10** |  | Vehicle |  | 0.6 ± 0.2 |  | 0.2 ± 0.1 |  | 0.9 ± 0.5 |  | 0.8 ± 0.4 |  | 0.3 ± 0.2 |  | 0.9 ± 0.4 |  | 0.0 ± 0.0 |
|  |  | L-NAC |  | 0.0 ± 0.0 |  | 0.1 ± 0.1 |  | 0.4 ± 0.3 |  | 0.4 ± 0.2 |  | 0.3 ± 0.3 |  | 0.6 ± 0.4 |  | 0.0 ± 0.0 |
|  |  | L-NACme |  | 0.0 ± 0.0 |  | 0.1 ± 0.1 |  | 0.1 ± 0.1 |  | 0.4 ± 0.2 |  | 0.2 ± 0.2 |  | 0.2 ± 0.1 |  | 0.0 ± 0.0 |

L-NAC, N-acetyl-L-cysteine (500 μmol/kg, IV); L-NACme, L-NAC methyl ester (500 μmol/kg, IV). NLX, naloxone hydrochloride (1.5 mg/kg, IV). WDS, wet-dog shaking. FPL, fore-paw licking. The data are shown as mean ± SEM. There were 9 rats in each group. There were no between group differences for any body weight value (P > 0.05, for all comparisons).

**Supplemental Table 5**

Body Temperatures and body weights at key points of control (no fentanyl) study

| **Parameter** |  | **Injections** |  | **Agent** |  | **Pre-Inj1** |  | **Pre-NLX** |  | **Post-NLX** |  | **Pre-NLX versus**  **Pre-Inj1** |  | **Post-NLX versus Pre-NLX** |
| --- | --- | --- | --- | --- | --- | --- | --- | --- | --- | --- | --- | --- | --- | --- |
| **BW, gram** |  | Inj1-5 |  | Vehicle |  | 336 ± 1.1 |  | 339 ± 1.2 |  | 338 ± 1.5 |  | +2.4 ± 0.6* |  | -0.8 ± 0.6 |
|  |  |  |  | L-NAC |  | 337 ± 1.2 |  | 340 ± 1.3 |  | 339 ± 1.3 |  | +2.9 ± 0.5* |  | -0.6 ± 0.6 |
|  |  |  |  | L-NACme |  | 335 ± 1.3 |  | 340 ± 1.1 |  | 339 ± 1.1 |  | +4.2 ± 0.5* |  | -0.3 ± 0.6 |
|  |  | Inj1-10 |  | Vehicle |  | 335 ± 1.4 |  | 341 ± 2.2 |  | 349 ± 2.3 |  | +6.3 ± 1.0* |  | -0.9 ± 0.7 |
|  |  |  |  | L-NAC |  | 336 ± 1.2 |  | 344± 1.9 |  | 343 ± 2.0 |  | +7.1 ± 0.9* |  | -0.3 ± 0.6 |
|  |  |  |  | L-NACme |  | 336 ± 1.3 |  | 343 ± 1.5 |  | 342 ± 1.8 |  | +7.6 ± 0.9* |  | -0.8 ± 0.8 |
| **BT, ^o^C** |  | Inj1-5 |  | Vehicle |  | 37.5 ± 0.08 |  | 37.4 ± 0.06 |  | 37.5 ± 0.06 |  | -0.04 ± 0.048 |  | +0.03 ± 0.08 |
|  |  |  |  | L-NAC |  | 37.5 ± 0.06 |  | 37.6 ± 0.07 |  | 37.5 ± 0.05 |  | +0.02 ± 0.05 |  | -0.03 ± 0.04 |
|  |  |  |  | L-NACme |  | 37.6 ± 0.07 |  | 37.5 ± 0.06 |  | 37.5 ± 0.06 |  | -0.01 ± 0.06 |  | -0.04 ± 0.07 |
|  |  | Inj1-10 |  | Vehicle |  | 37.5 ± 0.06 |  | 37.6 ± 0.04 |  | 37.6 ± 0.05 |  | +0.07 ± 0.04 |  | +0.01 ± 0.07 |
|  |  |  |  | L-NAC |  | 37.6 ± 0.05 |  | 37.6 ± 0.06 |  | 37.7 ± 0.07 |  | +0.03 ± 0.05 |  | +0.01 ± 0.05 |
|  |  |  |  | L-NACme |  | 37.6 ± 0.05 |  | 37.6 ± 0.06 |  | 37.6 ± 0.05 |  | -0.03 ± 0.04 |  | +0.01 ± 0.08 |

BW, body weight. BT, body temperature. L-NAC, N-acetyl-L-cysteine (500 μmol/kg, IV); L-NACme, L-NAC methyl ester (500 μmol/kg, IV). NLX, naloxone hydrochloride (1.5 mg/kg, IV). The data are shown as mean ± SEM. There were 9 rats in each group. *P < 0.05, significant change from initial body weight (Inj1). There were no between group differences for any body weight value (P > 0.05, for all comparisons).

**Supplemental Table 6**

Body weights and body temperatures at key points of control (no fentanyl) study in which L-NAC and L-NACme were introduced with co-injection 6 of vehicle.

| **Parameter** |  | **Treatment groups** | | | | |
| --- | --- | --- | --- | --- | --- | --- |
| **Body Weight, grams** |  | **Vehicle** |  | **L-NAC** |  | **L-NACme** |
| Pre |  | 336 ± 1.0 |  | 337 ± 1.4 |  | 336 ± 1.3 |
| Post-Inj 5 |  | 341 ± 1.1 |  | 341 ± 1.5 |  | 343 ± 1.5 |
| Post-Inj 10 |  | 345 ± 1.0 |  | 346 ± 1.7 |  | 350 ± 1.4 |
| Post-NLX |  | 344 ± 1.2 |  | 345 ± 1.6 |  | 349 ± 1.6 |
| Δ1. Post-Inj 5 *vs* Pre |  | +4.1 ± 0.4* |  | +4.4 ± 0.5* |  | +6.7 ± 0.6* |
| Δ1. Post-Inj 10 *vs* Pre |  | +8.3 ± 0.8* |  | +9.0 ± 0.6* |  | +13.2 ± 0.6* |
| Δ3. Post-Inj10 *vs* Post-Inj 5 |  | +4.2 ± 0.7* |  | +4.8 ± 0.5* |  | +6.6 ± 0.6* |
| Δ4. Post-NLX *vs* Post-inj 10 |  | -0.8 ± 0.5 |  | -1.0 ± 0.7 |  | -1.1 ± 0.7 |
| **Body Temperature, ^o^C** |  | **Vehicle** |  | **L-NAC** |  | **L-NACme** |
| Pre |  | 37.5 ± 0.06 |  | 37.4 ± 0.06 |  | 37.5 ± 0.07 |
| Post-Inj 5 |  | 37.5 ± 0.05 |  | 37.4 ± 0.03 |  | 37.4 ± 0.06 |
| Post-Inj 10 |  | 37.6 ± 0.08 |  | 37.5 ± 0.05 |  | 37.5 ± 0.05 |
| Post-NLX |  | 37.6 ± 0.12 |  | 37.6 ± 0.10 |  | 37.6 ± 0.09 |
| Δ1. Post-Inj 5 *vs* Pre |  | +0.02 ± 0.06 |  | -0.02 ± 0.08 |  | -0.03 ± 0.04 |
| Δ1. Post-Inj 10 *vs* Pre |  | +0.04 ± 0.05 |  | +0.06 ± 0.03 |  | +0.02 ± 0.05 |
| Δ3. Post-Inj10 *vs* Post-Inj 5 |  | +0.02 ± 0.06 |  | +0.08 ± 0.06 |  | +0.06 ± 0.06 |
| Δ4. Post-NLX *vs* Post-inj 10 |  | +0.04 ± 0.06 |  | +0.06 ± 0.07 |  | +0.07 ± 0.06 |

NLX, naloxone hydrochloride (1.5 mg/kg, IV). L-NAC, N-acetyl-L-cysteine (500 μmol/kg, IV); L-NACme, L-NAC methyl ester (500 μmol/kg, IV). The data are presented as mean ± SEM. There were 9 rats in each group. *P < 0.05, significant change from Pre-values. Note that there were no between-group differences for any parameter (P >0.05, for all comparisons).
